# A balancing act: navigating the nuances of co-production in mental health research

**DOI:** 10.1186/s40900-024-00561-7

**Published:** 2024-03-07

**Authors:** Sophie Soklaridis, Holly Harris, Rowen Shier, Jordana Rovet, Georgia Black, Gail Bellissimo, Sam Gruszecki, Elizabeth Lin, Anna Di Giandomenico

**Affiliations:** Department of Education, Centre for Addiction and Mental Health, 1025 Queen St. West, Toronto, ON M6J 1H1 Canada

**Keywords:** Co-production, Values, Power, Vulnerability, Participatory action research, Recovery College

## Abstract

**Background:**

In the context of mental health research, co-production involves people with lived expertise, those with professional or academic expertise, and people with both of these perspectives collaborating to design and actualize research initiatives. In the literature, two dominant perspectives on co-production emerge. The first is in support of co-production, pointing to the transformative value of co-production for those involved, the quality of services developed through this process, as well as to broader system-level impacts (e.g. influencing changes in health system decision making, care practices, government policies, etc.). The second stance expresses scepticism about the capacity of co-production to engender genuine collaboration given the deeply ingrained power imbalances in the systems in which we operate. While some scholars have explored the intersections of these two perspectives, this body of literature remains limited.

**Main text:**

This paper contributes to the literature base by exploring the nuances of co-production in health research. Using our mental health participatory action research project as a case example, we explore the nuances of co-production through four key values that we embraced:

1. Navigating power relations together
2. Multi-directional learning
3. Slow and steady wins the race
4. Connecting through vulnerability

**Conclusions:**

By sharing these values and associated principles and practices, we invite readers to consider the complexities of co-production and explore how our experiences may inform their practice of co-production. Despite the inherent complexity of co-production, we contend that pursuing authentic and equitable collaborations is integral to shaping a more just and inclusive future in mental health research and the mental health system at large.

## Plain Language Summary

**Background:**

In the context of mental health research, co-production is a process where people with lived experiences, those with academic or professional experience, and people with both of these perspectives collaborate to design and actualize research initiatives. In the literature, there are two main opinions about co-production. The first opinion is that co-production is beneficial for those involved, improves the quality of services, and can also have impacts at higher system levels (e.g. influencing changes in health system decision making, care practices, government policies, etc.). The second opinion is doubtful that co-production has the ability to foster authentic collaboration because of the differences in power between academic and health systems. Even though some scholars have looked at both opinions, there is not a lot of research on this.

**Main Text:**

This paper contributes to the literature base exploring the nuances of co-production in health research. Using our mental health participatory action research project as a case example, we explore the nuances of co-production through four key values that we embraced:

1. Navigating power relations together
2. Multi-directional learning
3. Slow and steady wins the race
4. Connecting through vulnerability

**Conclusions:**

By sharing these values and associated principles and practices, we invite readers to consider the complexities of co-production and explore how our process may inform their engagement with co-production. We argue that pursuing authentic collaborations is key to shaping a more just and inclusive future in mental health research and the mental health system at large.

## Background

Co-production in mental health research involves people with lived expertise (PWLE) of accessing mental health services, those with professional or academic expertise, and people who have both of these perspectives collaborating to design and actualize research initiatives [1, 2]. Authentic co-production moves beyond a consultation model to recognize PWLE as experts, situating them as equal partners from the very beginning of an initiative [3, 4].

In the broader health literature, two dominant perspectives on co-production emerge. The first argues that co-production has the potential to make meaningful, transformative change. Oliver et al. [5] describe four primary arguments that endorse this view: (1) Co-produced research and programs are more likely to be impactful; (2) Co-production improves the quality of research and programs; (3) Co-production processes bring about transformation by dismantling power imbalances among people with various forms of knowledge and expertise; and (4) Co-production is a political act aimed towards dismantling negative stereotypes (e.g. sanism—a form of oppression that stems from prejudice against people with mental health conditions) and reshaping attitudes to drive more just and impactful research and services.

The second perspective expresses scepticism about the capacity of co-production to engender genuine collaboration [5, 6]. This position posits that equitable partnerships between PWLE and those with professional expertise are impossible because power imbalances are inextricably ingrained in the systems in which we operate. As a result, under the guise of co-production, tokenistic engagement occurs, thus reinforcing depoliticisation (i.e. hiding the influence of political factors) [7, 8]. Given that power imbalances underpin and remain pervasive in healthcare and academic systems, this position argues that truly equitable relationships cannot be achieved through co-production and the co-optation of lived expertise is inevitable [7, 9]. This standpoint promotes the idea that PWLE should organise outside of healthcare and academic systems to influence social and systemic change [7].

Although some scholars have explored the nuances at the intersections of these two perspectives [10, 11], this body of literature remains limited. We, the authorship team, are committed to the practice of co-production and to simultaneously challenging the dominant culture of paternalism, sanism, tokenism, exclusion, and oppression. Because we work in a psychiatric hospital setting, we will be describing our practice within the mental healthcare system and for mental health research. We strongly believe in the tenets of co-production (the first position) while also recognizing and engaging with the challenges that working equitably in hierarchical systems entail (the second position). In this paper, drawing on our experiences from a participatory action research (PAR) project, we contribute to the literature base by exploring the nuances of co-production in mental health research.

### The current paper

We are often asked how we *do* co-production. More specifically, people want to know how we navigate issues of power and privilege. To address this question, we engaged in a reflexive practice of documenting our process. We explored, recorded, and analysed our co-production processes, which provided the basis for this process paper. Through this reflexive process, our group came to understand how what we *do* is rooted in who we *are*, including the shared values we have developed as a group. Using our PAR project as a case example, we explore the nuances of co-production through four key values our team engaged with: (1) Navigating power together; (2) Multi-directional learning; (3) Slow and steady wins the race; and (4) Connecting through vulnerability. During one of our brainstorming sessions, we focused on determining the narrative we wanted to share in this manuscript. As part of this process, each of us crafted storybook-style reflections that outlined our journey. We extracted epigraphs from these reflections that best described our story and values and have used these to introduce each of the seven sections below. It is important to highlight that the values we describe are interconnected and interdependent, entangled in a complex web rather than existing as isolated components. These values intersect and influence each other, often blurring the lines between them. While we have endeavoured to present a linear description of our co-production values, co-production itself is non-linear and inherently iterative. For example, you will notice how many of the practices we employ align with multiple values, thus emphasising the intricate network of the values informing our actions and processes. Furthermore, the entwined nature of these guiding values means that they must be considered as a connected whole and that attempting to dissect them or identify which is the *key* value negates the essence of co-production. However, we also acknowledge co-produced research is contextual. Therefore, while we contend that the values we discuss are deeply important to undertaking co-produced research, we also acknowledge our experience was highly context-specific. Consequently, the specifics should be tailored and adapted based on the unique context of one’s own co-production work.

Our primary intention is to share our reflections on our organic and iterative process of co-production, along with our reflections. We encourage readers to join us in challenging established dominant discourses that dictate what knowledge is valuable, legitimate, and ‘counts.’ Refraining from a theoretical inquiry into co-production was a deliberate choice as numerous scholars have provided robust theoretical framings of co-production [12, 13]. Our aim is to operationalize the process of co-production through a case study example of how equitable and just principles can be implemented into collaborative research among the usual restrictive conventions that are embedded in the academic and health systems in which we operate.

By sharing our values and associated principles and practices in this paper, we invite you to consider the complexities of co-production and explore how our experiences may inform your collaborative practices. Moreover, we hope to illustrate that pursuing authentic and equitable collaborations in hierarchical systems is a worthwhile endeavour.

### Our team

*Sweet. Smart. Complete… There is no better team I would rather have.*

Collaborative work often involves people taking on specific roles depending on their areas of expertise. For instance, people are often categorised into certain roles such as researcher, lived experience advisor, or clinician. In contrast, our team consists of people who represent diverse disciplines, areas of focus, and learned/lived expertise, each with intersectional and complex experiences. Thus, we embody a relational approach which recognizes that research “is inevitably based on who we are, how we come to each other as researchers and/or the subjects of research, and the individual, unique, and personal nature of human relationships, including research relationships” [14 p. 12]. Rather than siloing people into one aspect of their identity (e.g. researcher or PWLE), we invite everyone to bring their whole selves to the research process, thus emphasising the full spectrum of our skills, strengths, and experiences. In Table 1 we provide positionality statements for each of us to give readers insights into who we are. As you read the statements, we invite you to consider whose voices are represented and whose are missing.

**Table 1:** Authors’ positionality statements

**Sophie Soklaridis** I am the daughter of Greek parents who immigrated to Canada. I grew up in Lourdes, Newfoundland, and Scarborough, Ontario. I hold assumptions and perspectives that are shaped by how I see/experience the world and how the world sees/experiences me. I work in an academic medical institution and recognise that it is a privileged site of knowledge production that has historically marginalised paradigms outside of the traditional biomedical model. I strongly believe in the importance of collaborating with lived-experience experts in research. My intent is to use my positional power to amplify their voices as experts who have invaluable knowledge to contribute to the research process	
**Holly Harris** I acknowledge the intersectional privilege/oppression that I experience on account of my identity. I am a white, middle-class, cisgender female with master’s-level education born and raised on stolen land. I identify as someone who is neurodivergent and a consumer/survivor of the psychiatric system. I am employed by a tertiary mental healthcare facility as a research coordinator and have been involved in RCs as a peer support specialist, peer facilitator, and research coordinator for six years. I leverage my lived experiences as a source of strength, resilience, and expertise to highlight the voices of those who have been historically silenced in the psychiatric system and academia. I am currently pursuing a doctoral degree in gender, feminist, and women’s studies focused on the integration of lived experience perspectives in psychiatric governance structures. I acknowledge that my lived, academic, and professional experiences influence the value I place on specific practices, ideas, places, and symbols, and on my interpretation of the data	
**Rowen Shier** I am a white settler, cisgender woman. I grew up in a low-income family and identify as someone with lived experience. I hold a master’s degree in social sciences focused on the intersection of policy and power relations. I recognise that my lens for engaging with this research process has been shaped by my various intersectional privileges and oppression. As a researcher, I am dedicated to anti-oppressive and community-engaged practices. I seek to make space for those who are commonly excluded from knowledge creation towards the goal of advancing social justice. Personally and professionally, I am committed to working in solidarity with people and communities to dismantle and challenge systems of oppression	
**Jordana Rovet** I recognize that my understanding of this subject is influenced by multiple intersecting aspects of my identity, including my lived and academic expertise. I am a white settler, middle-class, cisgender female with a master’s degree in social work. I have spent the last 10 years working in the area of mental health and substance use, supporting projects led by people with lived experience. I am currently employed by a large mental healthcare hospital as a coordinator, where I am directly involved in the design, implementation and evaluation of an RC. My positionality informs the lens I bring to the data and the ways in which I interpret the experiences of others	
**Georgia Black** I am a white, cisgender female who immigrated to Canada from Scotland in 2019. I have an undergraduate degree in psychology and have worked with populations that experience elevated rates of health inequity. While I am not involved in the design or implementation of RCs, I am involved in related research and have been part of the current project from its initiation. My professional background is underpinned by my personal experience of accessing and navigating mental health services. I acknowledge how both my *insider* and *outsider* status have shaped my approach to this research, including my perception of our team values; the writing I contributed to this paper; and my interpretation of what should be included during editing	
**Gail Bellissimo** I identify as a white middle-class, cisgender female. I acknowledge my social location and associated privileges, as well as experiences of stigma. I have spent eight years engaging in research in the areas of chronic health, mental health, patient-oriented research, and service user education. I have also been involved in the co-design of an RC for a large mental health care hospital. I seek opportunities to create inclusive and safe spaces by removing barriers to allow for capacity building, mentorship, education, and peer support for the voices that are denied access due to discrimination and biases	
**Sam Gruszecki** I identify as a white cis-gendered middle-aged male. I work as a coordinator for an RC and am an employee of the organisation that employs many of the people involved with this paper. This is part of my work. I also had collegial and community-based experience with most of these people before this work began. Some of my lived experience includes navigating anti-Semitism, neurodivergence, multiple diagnoses, and experiences with poverty, as well as being the child of an immigrant, navigating services, and lacking post-secondary education. I have been involved in RC work, funded through major hospitals, as a peer support specialist, lead peer and coordinator since 2014. My experiences in research are relatively limited and I continue to learn along the way	
**Elizabeth Lin** I am a non-white, middle-class female with a doctoral degree and have dealt with barriers for non-whites in more than one country. I have been employed by a tertiary mental healthcare facility as a research scientist for over 30 years and have an extensive track record in traditional health services research using quantitative and large survey methods. I am new to RC research, and this is the first project about RCs that I have been involved in. My interactions with individuals who have been part of RCs have largely been with this team and involve creating our co-productive process, writing manuscripts arising from this project and attending administrative meetings where RC students, administrators, or facilitators may also be present. My role in this project included contributing specific expertise on conducting scoping reviews and editing manuscripts for scholarly journals. My perceptions are very much influenced by my upbringing and the cultural and social context that I grew up in and had to navigate to gain my education and my current occupation	
**Anna Di Giandomenico** This project is my first experience with conducting research related to RCs. I have conducted patient-partnered research with a pan-Canadian diabetes research organisation and am an author on an academic publication related to that work. I have been a student in RCs for five years, a member of an RC course and programming committee for three years, and recently became a founding member of an RC research committee. I have a bachelor of arts degree in psychology and early childhood educator certification. Leveraging my education and lived experiences as an RC student in this project has been a positive experience for me	

### Our project

*Once, not so long ago, when a plague had swept the land, a group of people came together, as part of a tri-council grant*.

In 2019, a Recovery College (RC) was established at the Centre for Addiction and Mental Health (CAMH), where we conduct our work. RCs are low-barrier mental health and well-being oriented programs rooted in the principles of peer support [15]. They support people in identifying and pursuing their goals through educational opportunities. A central principle of the RC model is co-production. The establishment of the RC provided the impetus to pursue RC research at our institution. In 2020, we received tri-council funding from the Canadian Institutes of Health Research as part of a Strategy for Patient-Oriented Research Catalyst grant. Our PAR project, entitled *Our Recovery, Our Outcomes: Co-Producing an Evaluation of Recovery Colleges*, brought together a group of researchers and PWLE to co-produce research that would inform the development of evaluation strategies for RCs [16]. The project comprised two phases. In phase one, we conducted a scoping review that explored the current literature on evaluation in RCs, with a specific focus on examining whether RC evaluations are being co-produced [6, 17]. In phase two, we used a PAR approach to conduct semi-structured interviews with 29 people who were involved with RCs in Canada. The qualitative interviews asked what participants valued most about RCs and we thematically analysed their views on how best to evaluate them [18, 19]. The methods and findings for the scoping review and qualitative study are detailed in separate manuscripts [17–19]. In addition to the research outlined above, part of the vision of the original grant was to build and solidify a team including key stakeholders in RCs with the aim of co-producing a future tri-council grant application. While the team completed the work outlined in the grant, the goals and priorities of the project evolved and expanded over time. The team pursued unexpected avenues of inquiry, experimented with creative forms of collaboration, and engaged with alternative forms of knowledge translation in addition to more *traditional* forms of research engagement. This manuscript is an example of an unexpected product from this work that emerged organically through our collaborative processes. Our collective core values underpinned all of our work, including the development of this manuscript.

## Main text

### Our values

#### Value 1: navigating power relations together

*Nothing’s perfect, that is true. It’s not easy to grapple with power, but by confronting the uncomfortable, this group has been allowed to flower*.

Definitions of power are a subject of ongoing debate in the literature. In contemporary power studies, three key categories emerge: power-over, power-to, and power-with [20, 21]. These expressions of power range from coercive, enabling, and coactive in terms of how they influence, empower, and control. It is important to emphasise that these types of power are not mutually exclusive, rather, they coexist in complex and multifaceted ways [21].

Through our co-production journey, we have come to understand power as inherently relational, embedded in and deployed through our relationships. In our experience, these relational aspects actively manifest across all three dimensions of power (power-over, power-to, and power-with) in nuanced and challenging ways. From this perspective, power is not static but dynamic, and while it can be wielded through our relationships, it can be balanced and shared through them as well [10, 22].

Navigating power relations is often regarded as one of the major stumbling blocks to co-production [10, 22]. Mental health research, at least within a North American context, takes place at the intersection of two systems that are deeply hierarchical in nature: mental healthcare and academia, both of which tend to privilege professional and academic knowledge while marginalising other ways of knowing (i.e. lived expertise) [11]. The power relations within our team are therefore nested within and connected to broader systems of power.

Instead of ignoring issues of power, actively engaging with power relations both as individuals and as a team was placed at the heart of this work. This meant that power had to be considered before we could conceptualise the grant. For example, in bringing together the research team, it was important to ensure that the team included more co-researchers (i.e. people invited to join the team as lived experience partners) than professional researchers. It was also necessary to think through the ways that power differentials could be tempered, if not eradicated; for example, by ensuring that people engaged as co-researchers were paid for their time.

Through our process, we realised that beyond developing a team of people with diverse perspectives and areas of expertise, it was crucial to examine the power inherent in each of our roles within the team. In traditional research, the principal investigator’s (PI’s) role is to ensure the project runs according to the proposal that was submitted and accepted by the funder. The PI is usually situated as the person who is most knowledgeable about the proposed research. In our project, the PI was responsible for bringing the outputs we were funded to produce to the team’s attention, but, in the spirit of co-production, it was up to the entire team to decide how we would go about delivering those results. There is a misconception that power-sharing or distributive leadership means there is no accountability and there is no leader who can be held to account if things go *wrong*. However, in coproduction, accountability is a shared activity. This translates into the PI not having ultimate control over how the research is conducted. Rather, the PI’s role is to facilitate shared decision-making. This can be a difficult paradigm shift for both the PI and funders.

Our team recognized early on the need to commit to a reflexive and continual process of critically examining how forms of power and privilege are reproduced, both within the team itself and through the research we are conducting. As a first step, our team created ways to actively engage with issues of power. We developed formal and informal channels that served this purpose, where issues around power could be explored either openly or privately. For example, after the first few weeks, the team made the joint decision to spend the last 10 to 15 min of our meetings debriefing. This was intended as a space where we could document our process and where members could voluntarily reflect on how the meeting went. Initially, debriefing felt stilted and superficial; however, over time, it became an extension of broader conversations we had engaged in as a team and offered a space where we could discuss issues related to power that may have arisen during particular meetings.

It is important to note that power differentials can often lead to conflict [5]. Instead of avoiding conflict or ignoring it, we viewed it as a learning opportunity that served a constructive purpose. Conflict among team members frequently led to a deeper understanding of each others’ perspectives and opportunities to learn from these diverse perspectives, and in some cases, generated innovative solutions. For example, when navigating power relations, our group evolved through conflict by calling each other *in* (as opposed to calling each other *out*), embodying accountability, reminding ourselves of our common purpose, and thus recentering ourselves as a team. This approach involved both the team’s willingness to support those who were having difficulties while remaining committed to our work and each other, as well as the willingness on the part of the individuals involved in conflict to reflect and share with the team (given it was safe to do so). What we have learned and developed in this process is a more complex way of interacting, which integrates team values with sensitivity and flexibility for individual needs. For example, we have developed a mutual recognition of when team members need space to self-reflect, as well as mechanisms for providing multiple ways for opposing opinions to be voiced, processed, and navigated (e.g. in a team meeting, in a private conversation with an individual team member). When conflict and differences of opinion are approached with the spirit of calling each other in, these conversations lead to critical epiphanies about how to work together more productively and compassionately.

Navigating power is an ongoing process as power shapeshifts. No sooner have you dismantled one small facet of power than it is reproduced in some other, often less easily detected, guise. It is therefore important to consider how practices that are introduced with the intention of increasing inclusion can unintentionally reinforce existing power hierarchies. For example, in co-produced research, one of the major ways that power is maintained and reinforced is through the act of writing [10, 23]. In our own project, we sought to develop an open writing process by using a shared online document platform that allows all team members to access, write, and review manuscript drafts together in real time. Although this approach is intended to deconstruct traditional writing practices, it still might exclude certain voices. There is the risk that a process such as group writing appears to democratise the writing process, but in actuality preserves the dominance of voices that may be more familiar with academic writing and are therefore able to write their ideas, comments, and revisions more quickly.

In our group, we have tried various ways to mitigate this risk by engaging in joint writing sessions with pauses for dialogue throughout, taking a flexible approach to writing timelines, and diversifying the ways our team can contribute to manuscript development. Some strategies we used included verbal feedback and creative activities, such as collaborative brainstorming, poetry, and story writing. However, practices that are effective in dismantling aspects of power can easily be undone further downstream. Staying with the example of writing, even with an inclusive *drafting* strategy, close attention needs to be paid during the *editing* process. There is a challenge in weaving together singular cohesive pieces of writing that reflect multiple voices while still adhering to a form and standard that is acceptable to the academic community. What this often means is that some voices are edited out in favour of voices that align with dominant academic discourses and forms of expression. Open drafting can therefore fall down through closed editing. In our work, we continue to seek to balance and discuss this tension as a team, with ample time set aside for group dialogue and contributions to edits, revisions, and the final version of the manuscript.

Getting to the point where power-sharing could be openly explored as a group took time, space, and above all, *trust*. We came to understand trust as the bedrock of navigating power relations. Team members had to trust that power would be shared, especially by those who held traditional positions of academic power through their role as researchers. Team members had to trust that any concerns they voiced about power would not simply be dismissed or glossed over. And team members had to trust that if power-sharing did not go as planned, the situation would be addressed directly and with sensitivity. That is not to say that these discussions always went perfectly. The discomfort of deeply engaging with power differentials means that our automatic response is often to retreat, become defensive, or “double down.” However, we found that by learning to lean into this discomfort and adopting an approach of *calling in* rather than *calling out*, we opened up space to explore power in a meaningful but balanced way with consideration for the safety of all team members.

Our team acknowledges that there is no endpoint at which power imbalances are completely neutralised. Instead, power relations are continually renegotiated and, by extension, so is power-sharing. The key for our team was that—wherever possible—we challenged and navigated the ebb and flow of power *together*.

#### Value 2: multi-directional learning

*As time went on the team made space for multi-directional learning. Through highlighting everyone’s unique expertise, the wheels definitely started turning*.

As a team, we grounded ourselves in a practice that we call multi-directional learning, which challenges the erroneous yet widespread assumption that engaging PWLE in research requires “cumbersome unidirectional capacity‐building whereby PWLE must be brought up to speed” [19 p. 1796]. In our project, multi-directional learning involves each team member simultaneously assuming the roles of teacher and learner while co-creating new knowledge at the intersection of diverse perspectives through the process. There is a richness in the variety of perspectives that each of us brings to the research project, and sharing one another’s knowledge is a privilege and a gift. Highlighting diverse ways of knowing and producing knowledge challenges the bias that has bred an intellectual culture that privileges biomedical approaches over other ways of knowing about mental health [24].

Multi-directional learning has taken two main forms in our research process: direct formal learning and informal organic learning. Direct formal learning refers to didactic learning sessions (e.g. lectures, structured presentations) followed by discussion. The main focus of these sessions was to create a shared knowledge base and group expertise. To support this, education sessions were a standing agenda item, with the sessions solicited by either asking team members for topics that they wanted to learn more about or for areas that they wished to share. Sessions included: presentations by team members on their own lived experiences navigating mental health challenges and systems; introductions to scoping review methods, evaluation, qualitative research, research software, epistemology, and ontology; the philosophy and history of RCs; the power of language; and a walkthrough of a complex analytic publication by a researcher team member. Each didactic presentation was followed by open discussion in which team members were able to ask questions, share reflections, and co-create new knowledge and ideas through collective exploration.

An additional form of direct formal learning was used to inform shared decision-making, particularly with regards to how to take advantage of or navigate external facilitators and barriers to the work that we wanted to do. For example, when we discussed the authorship order for one of our manuscripts, a team member who is a senior scientist explained what different positions in authorship order typically indicate about people’s contributions, what the implications are for funding, and what responsibilities lead authors have. Other team members shared their experiences (both good and bad) from other projects about how authorship was determined. We had honest discussions about the incongruence between our highly collaborative process and the hierarchical underpinnings of authorship lists. We collectively decided how to navigate authorship decisions in light of this incongruence. This is a process that we repeat for every manuscript and report we tackle.

Informal, organic learning opportunities also became possible through the culture of multi-directional learning that we fostered. Informal, organic learning refers to the ways in which, through dialogue, relationship building, and navigating differences in opinion, our group formed a cohesive identity and set of values, and generated new ideas and possibilities. One of the conditions which fostered opportunities for informal learning was mutual trust. We open every meeting with a check-in where team members share how they are doing, challenges they are experiencing, successes they want to celebrate, and anything else that is going on in their lives that they would like to communicate. Unlike in conventional meeting check-ins, team members share both work-related and personal updates. This allows us to get to know each other, understand each other’s experiences and perspectives, find things in common, and become aware of our unique strengths and interests.

For our group, check-ins are a routine yet informal organic practice that has stimulated many ideas for our work. For example, in some check-in stories, team members have shared their gardening woes and ongoing trials and tribulations with garden pests. When we were discussing the creation of an art piece [25], we decided to use a gardening metaphor to represent our process of co-production. The tone set by the check-ins and the importance we placed on building trust and nurturing relationships supported several other critical dynamics in terms of informal learning. An important one was that team members gradually felt more comfortable asking questions. Initially, several members would preface their questions by apologising for their self-identified lack of expertise or they would be hesitant to talk. But with consistent mutual support, those who initially believed they had little to contribute to certain conversations due to a perceived lack of subject expertise were able to reflect on unrecognised and untapped skills and bring them forth.

Our group also engaged in informal learning by doing. As described earlier in relation to power, we co-created a joint writing process that has resulted in an unexpectedly energetic and freeing experience where we feed off each other in real time and produce creative and frequently unexpected results. Our team identifies a topic for a manuscript, brainstorms using Google Jamboard, and co-designs a manuscript outline in Google Docs. Members then assign themselves to write or co-write specific sections of the manuscript, and then we collectively edit the manuscript in Google Docs before we submit it for publication. After writing and revising several manuscripts and presentations using this method, we have become surprisingly efficient. Given the response to our papers and presentations, this process is not only inclusive and efficient but often seems to produce valuable results in the external world’s judgement (as well as our own).

Multi-directional learning is made possible through flexible timelines. Having the time and space to explore tangents and engage in complex discussions has fostered creativity and freedom of thought [24] that becomes possible through the authentic engagement of people with various perspectives. The time needed for multi-directional learning is a worthwhile investment and creates opportunities for new knowledge and innovation.

#### Value 3: slow and steady wins the race

*The start was slow and hesitant, the course felt a little unsure.*

Our co-produced research necessitated a Gestalt shift in project time management. We quickly learned that unlike *traditional* approaches which prioritise rigid timelines, strict deadlines, and productivity outputs, meaningful collaboration meant that we had to embrace *flexibility*. We approached this in a twofold manner: pace and space. First, we adopted a slowed-down and adaptable pace to our work. In lieu of rigidity, we learned to approach timelines and deadlines loosely as much as possible, giving ourselves grace and permission to be human. Second, in alignment with our commitment to co-production and ensuring everyone’s voice and unique expertise was represented, we provided space for deviation from our original plans. Although embedding room to *get off track* may sound antithetical to the goals of research, in practice, it facilitated a deeper connection among team members and gave us the freedom to explore creative avenues for knowledge translation.

This freedom to explore also indicated that we valued the importance of slowness throughout our work. For us, slowness was a deliberate and political strategy towards creating a more democratic approach to co-producing knowledge. We recognized that not everyone experiences time in the same way. Since it takes time to generate knowledge, it was necessary to acknowledge that some people had more time for thinking and writing than others. For us, taking things slow was one strategy for creating an environment where individuals on the team had more equitable access to time. When co-producing knowledge, taking things slowly facilitated sustained engagement that moved beyond tokenistic involvement of lived experience knowledge and expertise [26]. Predictably, the shift in perspective was neither immediate nor without its challenges. It took *time* to both internalise and actualize the centrality of this approach to genuine co-production. We would be remiss not to acknowledge that early in the project we received a COVID-19 extension from our granting agency. This provided us an additional year to conduct our research and served as a gift of time that allowed us to wholeheartedly implement a gradual and more deliberate stride. Given the time-intensive nature of conducting meaningful patient-oriented research and PAR, we urge funding bodies to provide longer timeframes for the successful execution of such research [27]. In our work, we committed ourselves to this slowed-down process by implementing three fundamental practices.

First, as noted above, we adopted a gentle and collaborative approach to timelines and deadlines. We designed them to be fluid, functioning more as a guidance mechanism than a strict plan of action. For example, we initially established an ambitious timeline for writing this paper due to the impending conclusion of our grant; however, as other academic and personal priorities emerged, we allowed ourselves leeway to extend the process. This approach fostered a collective sense of accountability among the team while upholding each member’s autonomy to participate in the project in whichever way best suited them.

A second fundamental practice was to schedule our weekly meetings for *two* hours, which gave us the opportunity to engage in meaningful discussion, conduct project tasks synchronously, explore tangents when valuable insights arose (as they so often did), and take breaks when required. The environment of our meetings—like the project as a whole—fostered a culture of flexibility, adaptability, and built in time for team relationship building. We made concerted efforts through check-ins to gauge the team’s collective frame of mind, enabling last-minute adjustments to meeting agenda items in order to meet members where they were at on a particular day. We embedded protected time for storytelling in each meeting, which allowed team members to get to know each other on a personal level. Given the pandemic context of our work, these moments of humanity and levity were integral to breaking down the barrier of virtual engagement and, ultimately, for building trust. Finally, these practices (flexibility, trust building, camaraderie) sought to recognize and validate the emotional labour that is necessary when conducting co-produced research, particularly for PWLE [26].

As our third fundamental practice, we adopted a *health and wellness comes first* mentality in both the overarching project and in individual meetings. In contrast to traditional academic culture which does not care who you are or what you are going through as long as you produce, we made space to be patient, kind, and empathetic to each other and ourselves. For example, we took breaks, ended meetings early, organised social activities, and exchanged personal experiences.

While we hold that the success of our work greatly depended on this temporal flexibility, we admit that for several of us, the reconceptualisation of academic time demanded by co-production was quite uncomfortable at first. Without mandated deadlines and prescribed project management mechanisms, how could our research be productive, efficient, or competitive? How could we determine task accountability? Without direct oversight and control by the team members holding institutional leadership positions, wouldn’t the project disintegrate into chaos? Spoiler alert: it didn’t. By relinquishing control to the collective team and embracing uncertainty, the payoff was unexpectedly fruitful.

While we advocate for a slowed down pace and the creation of space wherever possible, we acknowledge the reality of contending with rigid external research deadlines (e.g. grant applications, conference submissions). In such cases, expeditious work often took precedence; for example, when the PI was invited to submit a commentary to the journal *Lancet Psychiatry*, the team mobilised and co-authored a manuscript in a matter of weeks [28]. Although the research community may purport to espouse patient-centredness and the inclusion of PWLE, the reality is that we operate in an environment with limited structural support to uphold these values and in which we are expected to get the work done as quickly as possible [27, 29]. But patients and PWLE are not peripheral to healthcare, and they should not be peripheral to healthcare research. Thus, during the moments in which we were compelled to churn out work, we endeavoured to reinforce collective team support and our shared humanity as a means to mitigate the demanding conditions. This is by no means a perfect solution, but it empowered us to navigate the inconsistencies between our values and the expectations ingrained within the systems in which we operate.

Research systems have historically prioritised—and continue to prioritise—a certain type of productivity, with success and impact metrics predominantly rooted in citation rates, impact factors, invited talks, and so forth [30]. Researchers are pressured to rely almost entirely on control and certainty in order to meet these traditional measures of success. Yet these metrics are biased, systematically silence socially marginalised and under-recognized groups, reinforce power structures, and ultimately fail to reflect the full spectrum of people’s meaningful contributions. We urge researchers to reflect on and challenge these constrained measures of success and the conventional pathways that they may have relied on. In any project where *voice* matters, we assert that we must reimagine our approach to time in order to create space to be heard, space to listen, and space to co-produce. Taking the time to do things together fosters a sense of shared ownership in the work. Things may have initially taken longer, but because we built trust and collective accountability, we were well-equipped and enthusiastic to meet our shared obligations in the face of impending deadlines as well as to pursue creative opportunities that were realised through our collaborative process. Specifically, our work has gained considerable traction and interest locally, nationally, and internationally.

At the local level, this work has become an important foundation for RC research at the CAMH and was a key factor in securing philanthropic funding for supporting a RC research portfolio. The funding enabled the establishment of a designated role for a Research Chair in Recovery and Equity-Focused Mental Health Education and a research subcommittee. The subcommittee is comprised of PWLE, people who access the RC at CAMH (the Collaborative Learning College), research professionals, evaluators, and RC staff. This group is responsible for collaborating with and advising other groups in the mental health community on matters relating to RC research and co-producing a research agenda, which includes the strategic directions for equity and recovery-oriented research.

On a national level, the findings from this project were shared with the Canadian RC Community of Practice (CoP), representing 150 members from over 50 organisations. This stimulated interest in exploring a co-produced evaluation process that is aligned across Canada supporting capacity building, developing a strong national voice, and advocating for the sustainability of the Canadian RC movement. As a result, facilitated by our PAR team, the national CoP has undertaken a co-production process to determine national RC metrics to pilot to catalyse this vision of alignment. The implementation plan for these metrics will be co-produced in an upcoming federally-funded workshop with RC stakeholders from across the country.

Internationally, we have established ourselves as key collaborators in the global RC movement. We have shared our work at numerous international conferences, including through invited keynote presentations. This has led to the establishment of strategic partnerships with other leaders in the RC movement through the form of research projects and strategic initiatives aimed at advancing the RC movement, peer support, and recovery-oriented practice and education.

#### Value 4: connecting through vulnerability

*Really the best outcome of all is the community we have made*.

Co-production, through its emphasis on challenging traditional power dynamics and centring lived experience, asks something unique of the people involved. It asks, and arguably requires, the willingness to incorporate the concept of vulnerability as part of creating a psychologically safe collaborative environment. To centre lived experience and foster genuine collaboration, we found it essential to put our ‘professional hats’ aside and connect as people first.

Outdated is the idea that good leaders and colleagues maintain stoic and unfeeling ways of being at work. Being vulnerable means not only accepting and expecting mistakes to happen, but when they do, the team comes together to learn from each other’s mistakes. We were honest with each other about when we were struggling, how we were feeling, and how, for many of us, this felt like navigating new terrain where we were given the opportunity and task of reimagining our professional identities and boundaries. This would not have been possible without trust.

We fostered trust as a group by prioritising getting to know one another as individuals. Diverging from the traditional expectation to leave our personal lives at home, we fostered an environment of honesty and openness, which allowed our relationships to move beyond those of work colleagues to become more like those of friends. Consequently, we created opportunities to celebrate and support one another through life’s ups and downs.

There was never an expectation of vulnerability, but over time, through a shared sense of non-judgment, we built a space for innovating and exploring ideas in new ways. The increased connection helped us to create the conditions under which we felt comfortable expressing alternative ideas, conflicting opinions, and points of concern. In these moments, we found that we were better able to navigate interpersonal conflict and disagreement from a place of understanding and mutual respect. We also felt safe presenting half-formed ideas, allowing us to build on one another’s train of thought and ultimately inspiring vital jumping-off points for our work. In prioritising our relationships and making space for vulnerability, we found not only that we were more productive as a group, but also that the connection we felt added to the richness and depth of the work we produced.

This is not to say it is always easy to be vulnerable. Academia is not typically a safe space to be vulnerable. This paper, for example, was extremely difficult to write because it reveals how our work is deeply ingrained in our subjectivities. Although we believe that our subjectivities and vulnerabilities bring richness to the work and to our team’s relationships, they can open us to criticism regarding potential biases and a perceived lack of rigour or trustworthiness in our work. Despite this, we have accepted the possibility that staying true to our values may also come at the expense of respectability, as traditionally defined by some academic communities. We continue to wrestle with this tension.

These complexities also find their way into our team’s engagement with one another. We have all had and continue to have moments of imposter syndrome, feelings of not belonging, feeling like we are outsiders, and feeling isolated. This work is emotionally charged because we have all been impacted by structures and systems that silence different aspects of our perspectives and identities in different ways. It is complicated. In our experience, these feelings crept up when we least expected it. What is important though is that we stood by each other when they did. We didn’t shy away from these conversations. When feelings started to bubble to the surface, we didn’t try to suppress them but rather muddled through the complex emotions together.

By challenging the notion that vulnerability is inappropriate or contradictory to professionalism, we made it possible to bring our full authentic selves to the table and foster connection through vulnerability.

## Conclusions

*Now the team is looking ahead to their next big idea and adventure!*

The values we have outlined are complex, interwoven, and dynamic, just as we, as humans, are. In providing our work as a case study, we hope to have prompted you, the reader, to reflect on what equitable partnerships entail and to explore how the values we have presented may significantly enrich and shape your collaborative endeavours.

At the paper’s outset, we described a discord between two differing views of co-production, noting how work within the intersection or synthesis of the two paradigms remains limited. Through our co-production process, we have successfully negotiated key tensions between the two standpoints and therefore offer our insights into how to navigate the nuances of co-production to engender meaningful, authentic, and productive relationships.

We recognize that co-production, collaboration, and equitable partnerships are a complex journey rather than a destination. Co-produced research is not an exact science, and there is no single recipe. By approaching this work with a recognition that we will never have all of the answers and a commitment to ongoing learning and accountability, we can actualize a more equitable and inclusive future in which people with lived expertise, those with academic or professional expertise, and people with both of these perspectives have equal seats and equitable voices at the table. We invite you to start where you are.

We acknowledge the complex emotions that emerge when we look back at the relationships we have fostered, the challenges we have faced, what we have created, what we have learned, and who we have become as individuals and as a team through this process. We share this work and our vulnerabilities because it is important. It is hard work to form and nurture equitable relationships in systems that are hierarchical and exclusionary in nature. But we fiercely argue that this is work worth doing. It is supposed to be hard and messy. The pursuit of equitable partnerships is a political act of system transformation. Working through challenges is where personal growth happens and the seeds of system transformation are planted.

When people are empowered to come forward and share their humanity as flawed, gracious, caring, and vulnerable human beings, we can create safer spaces in which to share expertise and leverage strengths. We are proud of what we have produced, but we are equally proud of who we were and who we were to one another while doing the work.

## Data Availability

Not applicable.

## Abbreviations

PAR: Participatory action research
PWLE: Person with lived expertise
PI: Principal investigator
RC: Recovery College
CAMH: Centre for addiction and mental health
CoP: Community of practice
